# Complementarity effects on tree growth are contingent on tree size and climatic conditions across Europe

**DOI:** 10.1038/srep32233

**Published:** 2016-08-30

**Authors:** Jaime Madrigal-González, Paloma Ruiz-Benito, Sophia Ratcliffe, Joaquín Calatayud, Gerald Kändler, Aleksi Lehtonen, Jonas Dahlgren, Christian Wirth, Miguel A. Zavala

**Affiliations:** 1 Forest Ecology and Restoration Group, Departamento de Ciencias de la Vida, Facultad de Ciencias, Universidad de Alcalá, Campus Universitario, 28871, Alcalá de Henares (Madrid), Spain; 2 Biological and Environmental Sciences, School of Natural Sciences.University of Stirling, FK9 4LA, Stirling, United Kingdom; 3 Department of Systematic Botany and Functional Biodiversity, Institute of Biology, University Leipzig (ULE, Germany); 4 Department of Biogeography and Global Change, Museo Nacional de Ciencias Naturales (MNCN-CSIC), C/José Gutiérrez Abascal 2, 28006 Madrid Spain; 5 Forstliche Versuchs- und Forschungsanstalt Baden-Württemberg (FVA, Germany); 6 Natural Resource Institute Finland (LUKE, Finland); 7 Swedish University of Agricultural Sciences (SLU, Sweden); 8 German Centre for Integrative Biodiversity Research (iDiv, Germany).

## Abstract

Neglecting tree size and stand structure dynamics might bias the interpretation of the diversity-productivity relationship in forests. Here we show evidence that complementarity is contingent on tree size across large-scale climatic gradients in Europe. We compiled growth data of the 14 most dominant tree species in 32,628 permanent plots covering boreal, temperate and Mediterranean forest biomes. Niche complementarity is expected to result in significant growth increments of trees surrounded by a larger proportion of functionally dissimilar neighbours. Functional dissimilarity at the tree level was assessed using four functional types: i.e. broad-leaved deciduous, broad-leaved evergreen, needle-leaved deciduous and needle-leaved evergreen. Using Linear Mixed Models we show that, complementarity effects depend on tree size along an energy availability gradient across Europe. Specifically: (i) complementarity effects at low and intermediate positions of the gradient (coldest-temperate areas) were stronger for small than for large trees; (ii) in contrast, at the upper end of the gradient (warmer regions), complementarity is more widespread in larger than smaller trees, which in turn showed negative growth responses to increased functional dissimilarity. Our findings suggest that the outcome of species mixing on stand productivity might critically depend on individual size distribution structure along gradients of environmental variation.

Growing evidence supporting a causal relationship between increased tree functional diversity and above-ground wood production^1^,^2^,^3^,^4^ reflects a major role of biodiversity on ecosystem functioning in forests^5^,^6^,^7^,^8^. Forests cover almost one third of the emerged lands and represent strategic resources for human societies so current species loss rates raise major challenges for human well being on Earth^9^. Consequences of species loss on forest productivity are however uncertain since diversity effects and the interactions with associated abiotic and biotic determinants are still poorly understood^10^. In particular, the role of population and individual tree level features such as size or age on the diversity-productivity relationship remain unclear^11^.

Classical experiments, mostly conducted on short-lived communities (i.e. grasslands, arthropod communities and microbial microcosms), point to niche complementarity, and the greater potential for species packing, as a chief mechanism by which the productivity of species mixtures is enhanced compared to the respective monocultures^12^ (i.e. overyielding). In forests, empirical evidence of a more intense exploitation of above-13 and below-ground resources^14^ in species mixtures also suggests a major role of niche complementarity. However, recent studies based on the use of different diversity metrics^2^,^7^,^15^ and modelling experiments^16^ are inconclusive as to where and when niche complementarity is a relevant mechanism driving diversity-productivity relations^17^.

To understand the functioning of long-lived communities an appreciation of their size structure dynamics is essential^18^. Trees have a continuous size development, which modulates their growth^19^, stand productivity^20^ and within-community interactions^21^. A different size implies niche differentiation (both between conspecifics or heterospecifics^22^) for resource uptake above- and belowground and so it could determine idiosyncratic growth responses along climatic and structural gradients^23^,^24^ thus driving population and community dynamics via tree-tree interactions^25^. Experimental evidence from tropical forest plantations has demonstrated that if biodiversity effects can be reduced to a neighbourhood effect, responses to biodiversity will result from the aggregated effects of local neighbourhoods within plots^26^. Accordingly, a tree-level approach may allow us to properly evaluate complementarity by accounting for individual tree attributes that can be critical in their response to the local environment^27^. In situations where strong interspecific competition is mostly established aboveground, size stratification combined with disparate light use strategies can lead to improved light-use efficiency and thus increased biomass packing and productivity^13^. In support to this idea, recent literature shows how tree size inequality in mixed communities enhances light-resource-use efficiency thus promoting positive diversity-productivity relationships through increased above-ground biomass packing^28^,^29^. In contrast, tree size inequality in monospecific forests has been shown to exert net negative effects on stand productivity due to strong intraspecific asymmetric competition^30^. Thus, size-structure dynamics and shifting tree-tree interactions during secondary succession are expected to alter the biodiversity-ecosystem functioning relationship^31^,^32^,^33^. Unfortunately, classical experiments are unsuitable for addressing long-term processes related with community size-structure dynamics at broad spatial scales^34^,^35^, and forest experiments are still at an early stage to provide solid evidence of mechanisms underpinning long-term diversity effects^36^.

Here, we investigated whether complementarity effects are contingent on tree size across the main climatic gradient of Europe from southern Spain to northern Scandinavian forests. Niche complementarity at the tree level would imply improved individual tree growth rates when growing in neighbourhoods surrounded by a larger proportion of functionally dissimilar neighbours. Although tree growth does not necessarily correlate in a one-to-one relationship with stand productivity, forest ecosystem production can, as a first approximation, be evaluated as the collective sum of individual tree productivity as individual tree growth plays a pivotal role in ecosystem functioning^19^. Specifically, we assessed for each focal tree the stand relative abundance (estimated as the proportion of the stand basal area) of functionally dissimilar tree species (POFT) (i.e. broad-leaved deciduous, broad-leaved evergreen, needle-leaved deciduous, needle-leaved-evergreen). Thereafter, we regressed focal trees growth against interactive effects of abundance of functionally dissimilar neighbours (POFT), focal tree size (SIZE), stand basal area (SBA) and potential evapotranspiration (PET). We evaluated whether positive growth responses to increased neighbourhood dissimilarity are modulated by tree size across stand structure (i.e. SBA as a proxy of the intensity of interactions) and climatic gradients (e.g. PET as a proxy of available energy). We expect that, in absence of strong belowground limitations, size asymmetric competition would favour complementarity effects according to size stratification of early- and late-successional species during secondary succession^13^,^32^. Large trees, in turn, may or may not experience significant positive effects from species mixing depending on whether belowground interactions result in facilitation^37^ or competition^38^. Recent findings in temperate forests suggest, in accordance with predictions of the Stress Gradient Hypothesis, that increasing frequency of positive plant-plant interactions due to enhanced stressed conditions might play a key role in driving interspecific interactions and thus higher biodiversity might lead to increased productivity^37^,^39^. Nonetheless, positive interactions represent only a fraction of complementarity effects and so positive plant-plant interactions might be obscured by pervasive interspecific competition even in less productive environments^40^.

## Results

The backward selection of predictor variables in the Linear Mixed Model suggested that one out of the four possible three-variable interactions (i.e. SIZE × POFT × PET) and two pairwise interactions (e.g. SIZE × SBA and SBA × PET) should be included in the fixed effects model (Table 1, see also parameter estimates in Appendix A Table A1). A random term with two components was included in the best supported model: species was included to affect the intercept and the slopes associated with POFT and SIZE; and plots nested in countries was included to affect the intercept parameter and the main effects of POFT (see random term selection based on information criterion in Table B1, Appendix B). Interestingly, the effects of SBA were not affected by the random structure ‘plots nested in countries’. The analysis of residuals indicates that the main assumptions of linear models were met (see Fig. A1a,b,c in Appendix A), and the pseudo-R^2^ pointed to a high goodness of fit, with more than 70% of variance explained by the final model (see Table 1).

**Figure 1:**
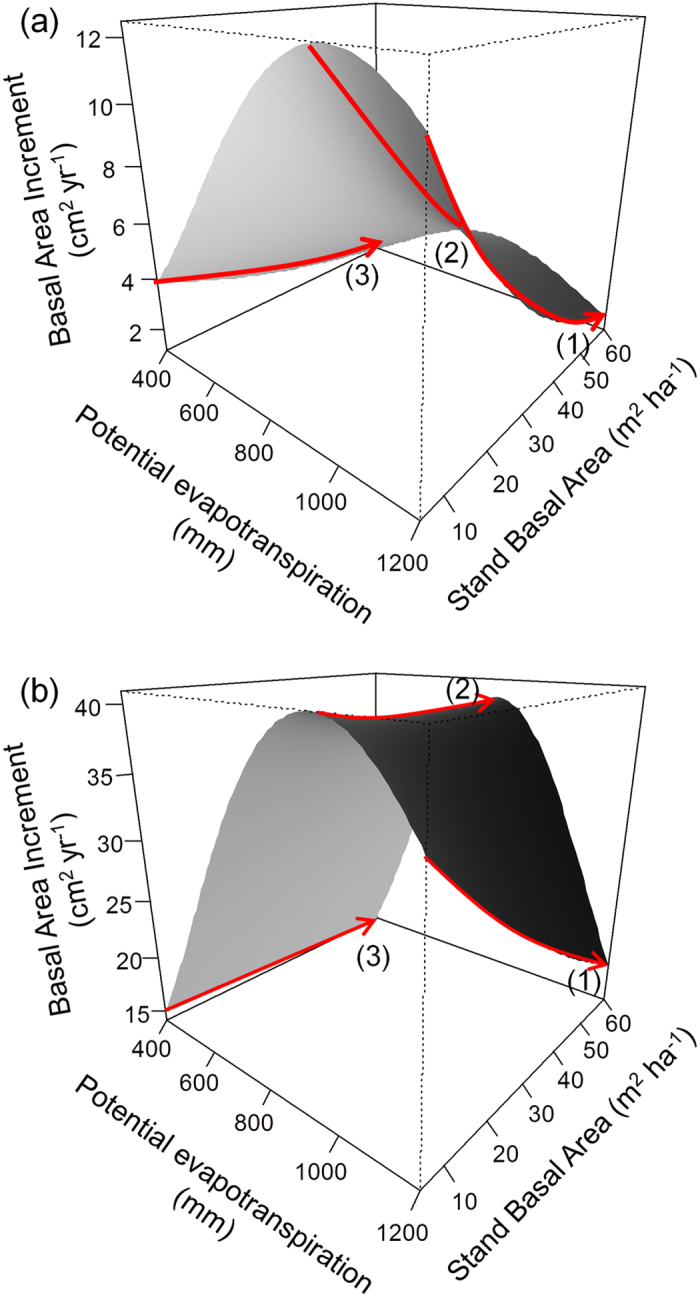
Predicted tree growth as function of potential evapotranspiration and stand basal area keeping size and the proportion of other functional types constant in mean values. Red arrows indicate the main trends of tree growth at low, intermediate and high PET values.

Firstly, variable selection pointed to significant interactive effects of PET × SBA and SIZE × SBA. Negative effects of SBA were pervasive along the entire PET gradient (Fig. 1) although they were notably stronger in smaller trees. Interestingly, the effects of POFT on tree growth were independent of SBA as shown by the results of the backward selection round#2, where the interaction SBA × POFT was finally discarded following the Bayesian information criterion (see Table 1).

**Figure 2:**
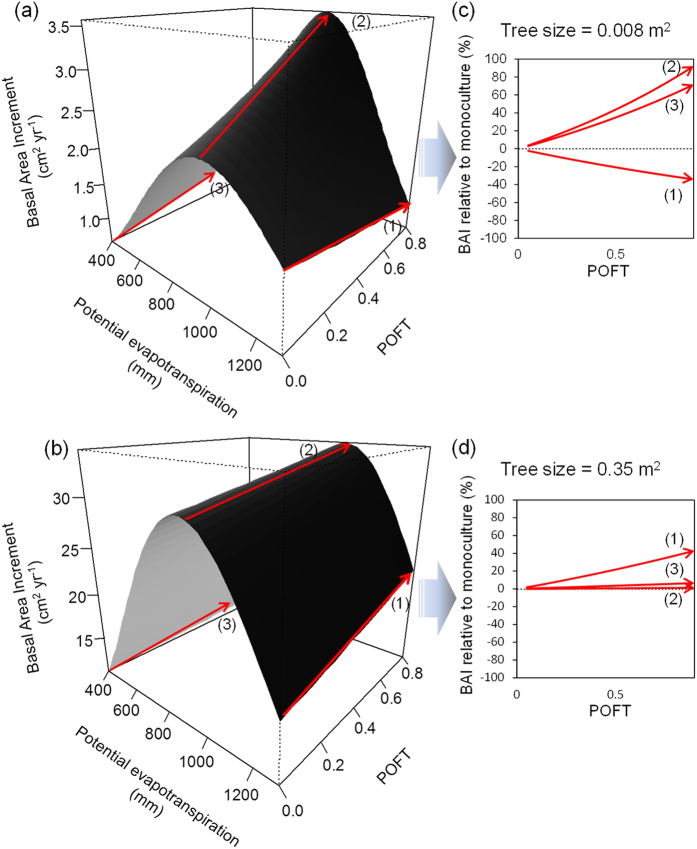
Predicted tree growth as function of potential evapotranspiration and the proportion of other functional types for small (**a**) and large trees (**b**) (basal area 0.008 m^2^ and 0.35 m^2^ respectively) keeping stand basal area in the mean value (25 m^2^ ha^−1^). Plots on the right represent predicted growth relative to growth in monoculture (%) for small (**c**) and large trees (**d**) keeping stand basal area in the mean value (25 m^2^ ha^−1^). Red arrows are indicative of the main trends of tree growth along low, intermediate and high PET values.

Secondly, we found that POFT effects on tree growth depend on tree size and the intensity and sign of this dependency change along the PET gradient. In particular, in areas of low PET (≈400–500 mm, boreal and alpine biomes), our results indicate complementarity effects on tree growth of small and large trees (Fig. 2a3,b3). Specifically, small focal trees (e.g. 10 cm DBH) surrounded by neighborhoods composed of 50% of other functional types can grow 44.4% more than similar focal trees from monospecific stands. A similar POFT scenario in large trees (e.g. 66 cm DBH) predicted tree growth rates to be only 0.68% higher in mixtures than monospecific stands. Under moderate PET values (i.e. 700–800 mm) the pattern is overall similar to that of boreal areas: i.e. complementarity tended to decrease notably towards sized trees which only grow 3.7% more when surrounded by neighborhoods composed of 50% of other functional types (see Fig. 2a2,b2). In similar conditions, smaller trees grew up to 37% more in mixtures than monospecific stands. Finally, growth responses to a greater POFT in Mediterranean latitudes shifted from net negative to positive as tree size increased (see Fig. 2a1,b1). Thus, a neighborhood composed of 50% of other functional types determines growth rates increments up to 21% compared to monospecific stands in large trees and growth reductions of 24% in small trees.

## Discussion

### Tree size and complementarity effects

We found tree size to be a critical factor driving complementarity effects in forests across Europe. This agrees with our expectations associated limiting resources along the large-scale PET gradient. Hence, complementarity effects can emerge from niche separation along spatial/temporal gradients of resource availability and thus tree size plays a pivotal role in determining resource use partitioning. In space, the combination of different light intercepting strategies and size stratification have been reported to significantly enhance resource yielding and biomass packing in mixed forest stands^41^,^42^. Partitioning of soil nutrient uptake through different degrees of fine root development can also trigger overyielding among dissimilar tree individuals although this poses major methodological challenges and uncertainties to identify^43^. Evidence from grassland studies suggests that interspecific differences in leaf phenology might determine temporal segregation of light capture and thus species coexistence^44^. Decoupled light use patterns associated with a different duration of the growing periods between young and old trees^45^,^46^,^47^ might explain size-dependent overyielding in species mixtures. This is the case of young evergreen trees in boreal-temperate forests where a seasonal decoupling of photosynthetic activity with adult broad-leaved trees might lead to complementary usage of light and thus coexistence^48^. Thus, differences in Specific Leaf Area, crown architecture and leaf phenology, and the subsequent light use strategies play a pivotal role in forest dynamics and tree development^49^,^50^,^51^. Projected onto a secondary succession scenario in temperate latitudes, species mixing is common in transitional stages when adult individuals of early succesional tree species coincide with juvenile individuals of late-succesional species and thus size stratification allows for biomass packing and overyielding.

### Tree size effects on complementarity across the energy availability gradient

In boreo-alpine forests (400–500 mm) our results indicated that strong complementarity effects on small individuals notably weakens with size development. Complementarity in nitrogen uptake^43^ and bidirectional transferences of soil carbohydrates via mycorrhiza^52^ have been reported as important mechanisms underpinning the positive diversity-productivity relationship in boreal forests. Interestingly, Cavard and cols^53^ showed how positive diversity-growth relationships in species mixtures disappear as tree communities mature. Soil nutrient dilapidation during post-fire secondary succession was invoked a plausible explanation for this reduction of complementarity effects in time. Recent findings in Europe suggest a comparatively minor role of functional dispersion on stand growth towards northern latitudes^15^ which is consistent with this successional scheme if we assume that larger trees have an exponentially higher contribution with stand productivity than smaller ones and thus functional responses of tree communities mostly resemble large-tree responses. Otherwise, a positive diversity-productivity relationship has been extensively reported in European and North American boreal forests in support of the idea that complementarity effects, although comparatively less important than other drivers of tree growth in these latitudes, are important determinants of forest productivity as well.

In temperate forests, complementarity effects depict a similar pattern to the one observed in boreal forests: e.g. strong complementarity effects decrease with tree development. Young broad-leaved deciduous trees are known to benefit from higher light availability when part of the forest overstorey is occupied by early successional conifers^32^. A recent review, however, highlighted the great number of potential factors that might alter the relative role of niche complementarity driving tree growth in temperate forests^54^. Moreover, results obtained from permanent plots in central Europe suggest that the lack of overyielding in temperate forests might be attributed to methodological factors associated with specific response variables and methods to compare pure and mixed stands^27^. In particular, accounting for crown biomass changes has been found to be decisive in detecting overyielding in temperate forests. Thus, forest inventory data may be limited to discern such fine-scale diversity effects.

In Mediterranean forests, our results point to strong complementarity effects in large trees, and contrary, negative effects of functional dissimilarity in small trees. Assuming that larger trees have a comparatively higher contribution to total stand biomass and stand basal area change, this finding is consistent with the existing literature posing significant positive diversity-productivity relationships in Mediterranean forests^3^,^7^. Complementarity effects in large trees have been associated with disparate light-use strategies and increased light-use efficiency^3^ and increased soil fertility^55^ due to enhanced litter quality. A positive diversity-productivity relationship in water-limited ecosystems has been also discussed in the theoretical framework of the Stress Gradient Hypothesis: e.g. increased abiotic stress conditions lead to increasing frequency and intensity of positive interspecific interactions^37^. Alternatively, insights from radial growth increments in mixed and monospecific pine-oak stands in Central Spain are consistent with the idea that stressed conditions due to soil water scarcity during intense droughts leave less room for complementarity effects in the Mediterranean^38^. Yet our results do not allow us to discern which mechanisms underlie the observed reversal, lacking complementarity effects in small trees could be attributed to inevitable trade-offs associated with adaptations to cope simultaneously with shade and drought^56^. Ecological studies on plant-plant facilitation in arid lands during the last two decades suggest that shade imposes an investment in aboveground parts (e.g. leaves) to increase light interception in the understory at the cost of exacerbated water losses due to higher shoot to root ratio^57^. Hence, size stratification is not an efficient way to optimize light interception, as in other biomes, because high evapotranspiration regimes expose understory tree individuals to water stress. It is important to note that in warmer Mediterranean areas, the dominant functional types are broad-leaved evergreen tree species and needle-leaved evergreen tree species. This implies that seasonal decoupling of leaf phenology is no longer a plausible complementarity mechanism in time so young/small conifer trees must overcome strong competition for light with adult evergreen broad-leaved trees. On the other hand, many species are resprouters and many of the smaller individuals are ramets which have better developed root systems and carbohydrate storages relative to suppressed saplings in pine stands. Complementarity among adult trees, in contrast, is less dependent on functional trade-offs associated with light and soil water limitations and disparate water-use strategies or root architecture may ameliorate intraspecific competition for soil water.

### Concluding remarks and future lines

Neglecting tree size and stand structure and assessing stand growth as an aggregated tree growth measurement might bias the interpretation of the diversity-productivity relationship in forests^29^ because growth responses of larger trees override those of smaller trees given the exponential relationship between growth and size^19^. Positive effects of tree diversity on stand growth in the Mediterranean^3^ and almost neutral-positive effects in temperate and boreal forests^14^,^15^ would mask the actual contribution of each individual tree. Thus, it is critical to consider tree size to properly evaluate diversity effects on tree growth throughout the course of tree development in different forest types across climatic gradients^29^,^54^,^58^. Future research efforts are needed to understand ecophysiological processes underpinning large-scale reversals in complementarity due to tree size. It is critical to understand how individual tree features such as size determine tree-tree interactions and how they influence secondary succession dynamics and ecosystem function. This would allow us to better understand the role of positive and negative interactions on size-structured community dynamics, and to establish more efficient conservation and management strategies based on community structure and species interactions rather than on individual species.

Our broad scale approach, however, lacks information on potential drivers of productivity such as soil nutrients, herbivory, disturbances and slight deviations from long-term climatic conditions at each sampling period might affect our findings since all these uncertainties might alter the diversity-productivity relationship. Given the impossibility of long-term experiments for exploring biodiversity-ecosystem functioning relationships in forests (at least for some years to come), we rely on improved large-scale data analyses and modelling. Therefore, further efforts are needed to improve forest inventory protocols and large-scale data harmonization to account for potential confounding drivers of productivity such as soil information, management, secondary succession and herbivory in forest ecosystems.

## Methods

### National forest inventory data: plot selection and focal species

**Figure 3:**
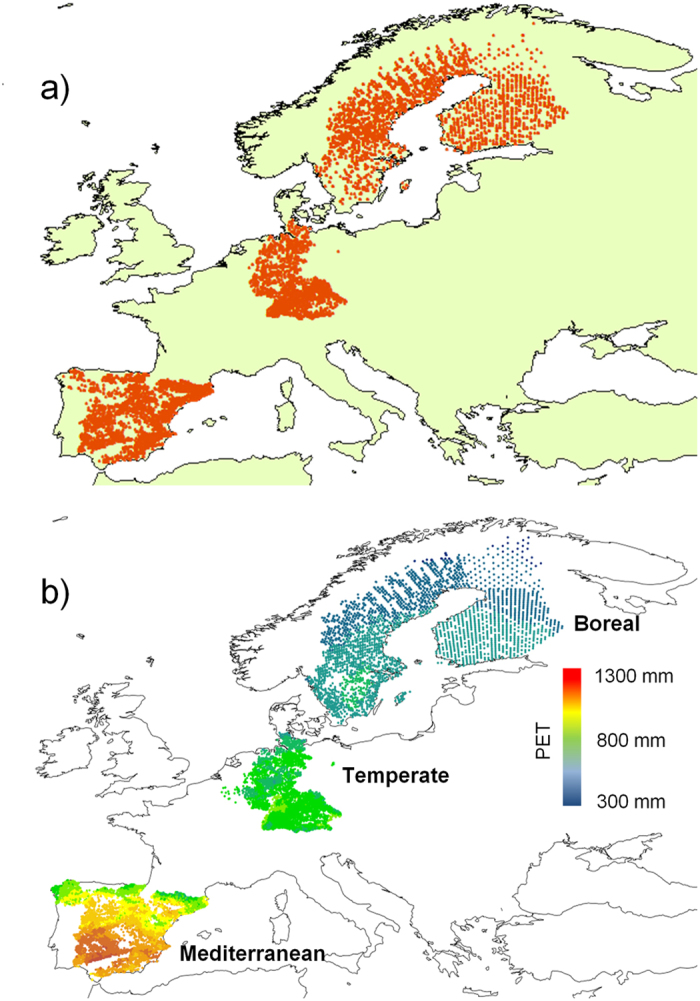
(**a**) Location of forest inventory plots across the study area (Europe) and (**b**) potential evapotranspiration (units) throughout the study area. Own preparation based on (**a**) plot coordinates included in the National Forest Inventories considered, and (**b**) Potential Evapotranspiration data available in CGIAR-CSI GeoPortal^60^ using software ArcGIS 13.0 by Esri (license University of Alcalá).

We used data compiled from the National Forest Inventories (NFIs) of five European countries (Finland, Germany, Spain, Sweden and Belgium -region of Wallonia, see a detailed description of each NFI in Ratcliffe and cols^15^). Permanent plots with two consecutive surveys and no evidence of tree harvesting before and within consecutive surveys were selected. From those plots, only trees >= 10 cm diameter at breast height (DBH) were included in the dataset to standardise tree selection between the different inventories. The 14 most representative tree species in terms of abundance and distribution were selected as focal trees: *Abies alba* (needle-leaved evergreen, temperate mountains), *Acer pseudoplatanus* (broad-leaved deciduous, temperate), *Betula pendula* (broad-leaved deciduous, boreal-temperate), *Betula pubescens* (broad-leaved deciduous, boreal-temperate), *Carpinus betulus* (broad-leaved deciduous, temperate), *Castanea sativa* (broad-leaved deciduous, temperate), *Fagus sylvativa* (broad-leaved deciduous, temperate), *Juniperus thurifera* (needle-leaved evergreen, Mediterranean mountains), *Picea abies* (needle-leaved evergreen, boreal-alpine), *Pinus halepensis* (needle-leaved evergreen, Mediterranean), *Pinus sylvestris* (needle-leaved evergreen, boreal-alpine-temperate), *Quercus pyrenaica* (broad-leaved deciduous, Mediterranean-temperate transition), *Quercus robur* (broad-leaved deciduous, temperate), and *Quercus ilex* (broad-leaved evergreen, Mediterranean). These species represent more than one third of the total stand basal area measured in the whole dataset and are the dominant tree species in at least one of the three principal forest biomes of Europe (i.e. Mediterranean, temperate and boreal). The final number of plots was 32,628 and the number of surviving trees 275,558 after removing dead trees or surviving trees with negative growth rates (7209 trees, 2.6%). Table 2 details the number of plots in the analysis and important plot-level statistics. Figure 3a illustrates the distribution of plots across the continent.

### Tree growth and complementarity

We assessed individual tree growth as the basal area increment (BAI) relative to the initial tree basal area and the number of years between consecutive surveys (i.e. annual basal area increment). Tree basal area in each inventory was calculated using dbh measurements as:


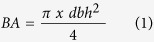


where dbh is the diameter at breast height (i.e. 1.3 meters above ground surface) in metres.

Complementarity effects (CE) at the tree level imply that any given tree will grow faster when, keeping biotic and abiotic sources of variability constant, its neighborhood is composed of a larger proportion of functionally dissimilar trees. With this in mind we assessed the proportion of the stand basal area (SBA m^2^ ha^−1^) occupied by functional types other than the focal tree (POFT) as follows:


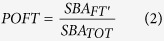


where SBA_FT′_ is the stand basal area of functional types different from the focal tree and SBA_TOT_ is the total stand basal area. Positive effects of this index on tree growth will be interpreted as complementarity effects while negative effects will be assumed prevailing interspecific competition. Yet this is of course an elementary approach to the complex world of niche complementarity metrics, its quantitative nature meets the rationale of the aforementioned definition of complementarity from the viewpoint of individual trees. We considered the four most general and divergent functional types regarding leaf phenology and morphology (i.e. broad-leaved deciduous, broad –leaved evergreen, needle-leaved deciduous, needle-leaved evergreen). Specific leaf area has been identified as a critical trait linked to species competitive ability (i.e. broad-leaved species tend to be more tolerant to competition and exhibit stronger competitive effects relative to needle-leaved species^49^). This classification is also coherent with functional differentiation based on wood density as needle-leaved trees tend to have lower wood density than broad-leaved trees and weaker competitive effects^49^. In general, differences in crown architecture, leaf phenology, photosynthetic activity, and root architecture between these general functional types have been shown to provide overyielding in boreal and temperate forests^14^,^41^,^42^.

### Abiotic and biotic drivers of tree growth

For each individual tree we noted the species identity and calculated the basal area of the tree (SIZE, mm) and the plot basal area (SBA, m^2^ ha^−1^) in which the tree resided. Mean climate (i.e. annual mean temperature (MAT, C), annual precipitation (AP, mm) and mean potential evapotranspiration (PET, mm)) for each plot were extracted from the WorldClim database^59^ and CGIAR-CSI GeoPortal^60^. Using PET and AP we assessed a water availability index (WAI) as:


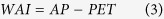


where negative values of this index denote water deficit because atmospheric water demand exceeds climatic inputs in rainfall. Because of the high correlation between PET and MAT (r = 0.93) and between PET and WAI (r = −0.71), we selected PET as an integrative variable that summarizes the two main climatic stress gradients in continental Europe, i.e. water deficit towards southern latitudes and low temperature towards northern latitudes and alpine areas (Fig. 3b).

### Data analysis

We fitted Linear Mixed Models (LMM) to model tree growth as function of SIZE, SBA, PET and POFT. Plot nested in country and species were included as two independent random terms in the model. Species was considered to affect the intercept parameter and the slopes associated with main effects of both POFT and SIZE under the assumption that (i) growth responses to an increasing proportion of other functional types might vary among the species considered in this study, and (ii) that growth patterns throughout the ontogeny might differ among species. Plot nested in country was considered to affect the intercept parameter and the effects of POFT on tree growth assuming that differences in plot size and sampling methodologies might affect the POFT assessment and thus the sensitivity of tree growth to this index. The model equation takes the form:


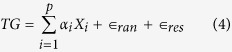


where *TG* is tree growth, 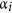 is the set of *p* parameters associated with the main and interactive effects of 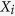 environmental variables, **∈**_*ran*_ is the variance component associated with the random terms, and **∈**_*res*_ is the residual normally-distributed error. We considered a fixed-effect term in the maximal model that included all the potential pair-wise interactions as well as the three variables interactions (i.e. SIZE × SBA × PET, SIZE × SBA × DIV, SIZE × PET × DIV, SBA × PET × DIV). SIZE was included in a log-transformed form and PET as a second order polynomial. Tree growth was log-transformed to meet normality and homogeneity of variance.

Model selection was conducted using a backward procedure of predictor variables starting with the maximal model^61^. We used the Bayesian Information Criterion (BIC) following the rule that net increments lower than 2 units of BIC associated with the elimination of any parameter in the maximal model determined the exclusion of the parameter from the final model^61^. We started with the selection of the three-variable interactions (round#1) and then tested the pairwise interactions (round#2) and so downwards the main effects of each predictor (round#3). A pseudo-R^2^ was assessed following Nakagawa and Schielzeth^62^. This index can be split into marginal and conditional effects, being the marginal R^2^ the goodness of fit of the fixed effect term only and the conditional R^2^ the goodness of fit of the whole model including the random term.

## Additional Information

**How to cite this article**: Madrigal-González, J. *et al.* Complementarity effects on tree growth are contingent on tree size and climatic conditions across Europe. *Sci. Rep.* **6**, 32233; doi: 10.1038/srep32233 (2016).

## Figures and Tables

**Table 1:** Fixed effects selection using the Bayesian Information Criterion (BIC).

Fixed effects selection	df	BIC	Delta BIC	R^2^-m	R^2^-c
Full-model (round #1)	33	610912.3	0		
SIZE × SBA × POFT (removed)	32	610889.8	−12.5		
SIZE × POFT × PET (removed)	31	610937.1	24.8		
POFT × SBA × PET (removed)	31	610900.8	−11.5		
SIZE × SBA × PET (removed)	31	610914.1	1.8		
Full-model (round #2)*	28	610891.0	0		
SIZE × SBA (removed)	27	614430.1	3539.1		
POFT × SBA (removed)	27	610885.3	−5.7		
PET × SBA (removed)	26	611043.7	152.7		
Best-supported model**	23	610885.3	0	0.367	0.705
null model (intercept only)	6	719146.1	108260.8		

We used a hierarchical backward selection of fixed terms starting with a full model that included all the possible three-variable interactions between the proportion of other functional types (POFT), tree basal area (SIZE, m^2^), stand basal area (SBA, m^2^ ha^−1^), and potential evapotranspiration (PET, mm). We tested the contribution of each interaction by removing them one at a time. R^2^-m is the marginal pseudo R^2^ (fixed effects only) and R^2^-c is the conditional pseudo R^2^ (including both fixed and conditional effects).

^*^The round#2 of the backward selection started with the best supported model obtained in the first round.

^**^Best supported model: SIZE × POFT × PET+SIZE × SBA + PET × SBA.

**Table 2:** Descriptive statistics of plot-level information for each National Forest Inventory (NFI) for the randomly selected populations of trees across Europe.

	Spain	Wallonia	Germany	Sweden	Finland	Total
No. plots	18 083	90	7 649	4 621	1 619	32 628
No. trees	146 799	668	38 620	48 902	15 888	275 558
No. species	11	4	9	5	6	14
PET (±SD)	1018.9 ± 163.2	715.8 ± 42.1	732.9 ± 49.1	508.8 ± 57.9	505.6 ± 42.5	812.2 ± 241.7
SBA (±SD)	9.4 ± 9.5	23.3 ± 12.5	25.6 ± 14.8	12.6 ± 10.7	10.3 ± 8.5	20.131 ± 13.0
MTBA (±SD)	0.06 ± 0.08	0.15 ± 0.18	0.09 ± 0.09	0.03 ± 0.03	0.03 ± 0.02	0.06 ± 0.08

Species ID (alphabetical order): *Abies alba* (1), *Acer pseudoplatanus* (2), *Betula pendula* (3), *Betula pubescens* (4), *Carpinus betulus* (5), *Castanea sativa* (6), *Fagus sylvativa* (7), *Juniperus thurifera* (8), *Picea abies* (9), *Pinus halepensis* (10), *Pinus sylvestris* (11), *Quercus ilex* (12), *Quercus pyrenaica* (13), *Quercus robur* (14). PET–Potential Evapotranspiration (mm); SBA–Stand Basal Area (m^2^ ha^−1^); MTBA– Mean Tree Basal Area (m^2^).
