# Erratum: Complementarity effects on tree growth are contingent on tree size and climatic conditions across Europe

**DOI:** 10.1038/srep46993

**Published:** 2018-06-04

**Authors:** Jaime Madrigal-González, Paloma Ruiz-Benito, Sophia Ratcliffe, Joaquín Calatayud, Gerald Kändler, Aleksi Lehtonen, Jonas Dahlgren, Christian Wirth, Miguel A. Zavala

Scientific Reports 6: Article number: 3223310.1038/srep32233; published online: 08 30 2016; updated: 06 04 2018

A Supplementary Information file containing Appendices A and B was omitted from the original version of this Article. This has been corrected in the HTML version of the Article; the PDF version was correct at time of publication.

